# The Mechanical and Biological Properties of Chitosan Scaffolds for Tissue Regeneration Templates Are Significantly Enhanced by Chitosan from *Gongronella butleri*

**DOI:** 10.3390/ma2020374

**Published:** 2009-04-20

**Authors:** Nitar Nwe, Tetsuya Furuike, Hiroshi Tamura

**Affiliations:** 1 Faculty of Chemistry, Materials and Bioengineering and HRC, Kansai University, Suita, Osaka 564-8680, Japan; E-Mails: nitarnwe@yahoo.com (N.N.); furuike@ipcku.kansai-u.ac.jp (T. F.); 2 Japan Society for the Promotion of Science (JSPS), Japan

**Keywords:** Characteristics, chitosan scaffolds, degradation, fungal chitosan, tissue regeneration template

## Abstract

Chitosan with a molecular weight (MW) of 10^4^ Da and 13% degree of acetylation (DA) was extracted from the mycelia of the fungus *Gongronella butleri* USDB 0201 grown in solid substrate fermentation and used to prepare scaffolds by the freeze-drying method. The mechanical and biological properties of the fungal chitosan scaffolds were evaluated and compared with those of scaffolds prepared using chitosans obtained from shrimp and crab shells and squid bone plates (MW 10^5^-10^6^ Da and DA 10-20%). Under scanning electron microscopy, it was observed that all scaffolds had average pore sizes of approximately 60-90 μm in diameter. Elongated pores were observed in shrimp chitosan scaffolds and polygonal pores were found in crab, squid and fungal chitosan scaffolds. The physico-chemical properties of the chitosans had an effect on the formation of pores in the scaffolds, that consequently influenced the mechanical and biological properties of the scaffolds. Fungal chitosan scaffolds showed excellent mechanical, water absorption and lysozyme degradation properties, whereas shrimp chitosan scaffolds (MW 10^6^Da and DA 12%) exhibited the lowest water absorption properties and lysozyme degradation rate. In the evaluation of biocompatibility of chitosan scaffolds, the ability of fibroblast NIH/3T3 cells to attach on all chitosan scaffolds was similar, but the proliferation of cells with polygonal morphology was faster on crab, squid and fungal chitosan scaffolds than on shrimp chitosan scaffolds. Therefore fungal chitosan scaffold, which has excellent mechanical and biological properties, is the most suitable scaffold to use as a template for tissue regeneration.

## 1. Introduction

Chitin is a copolymer of *N-*acetylglucosamine and glucosamine residues linked by β-1,4-glycosidic bonds. Chitosan is the deacetylated form of chitin. The names chitin and chitosan are operational terms, which are not precisely defined. Chitin usually refers to a copolymer with a degree of acetylation (DA) of more than 40% [i.e., degree of deacetylation (DD) of less than 60%] and insoluble in dilute acids. The name chitosan is used for a copolymer with less than 40% DA (i.e., more than 60% DD) that, in most cases, will be soluble in dilute acid. Fungi synthesize chitin and chitosan in their cell walls, while the shells of crabs and shrimps and the bone plates of squids and cuttlefish are composed of chitin only [1,2,3,4,5,6,7]. Pure chitosan is non-toxic, free of antigenic effects, biocompatible, biodegradable and polar [8,9,10,11]. It has been used to prepare a variety of forms such as powders, hydrogels, fibers, membranes, beads and porous scaffolds that have been tested in many medical and biological applications [12,13,14,15]. For tissue engineering applications, chitosan scaffolds have been prepared by the freeze drying and freeze gelation methods and by a 3-axis robotic arm dispensing system and their mechanical and biological properties have been characterized [8,13,15,16,17,18,19,20,21].

The mechanical and biological properties of scaffolds such as pore size, water absorption, sensitivity to lysozyme degradation, mechanical strength and cellular activities are important parameters for the application of the scaffolds in tissue engineering and for organ substitution. The mechanical and biological properties of chitosan scaffolds depend on the properties of chitosan, such as molecular weight, DA and degree of crystallinity, and on the scaffold preparation method used [8,20,22,23,24,25,26,27,28]. The lower DA chitosan scaffolds have smaller pore sizes, ranging from 50 to 100 μm, greater mechanical strength, moderate water absorption properties and higher cellular activities than higher DA chitosan scaffolds [18,20,22,25]. The lysozyme degradation rate of a chitosan scaffold is inversely related to the molecular weight and degree of crystallinity of the chitosan and proportionally related to its DA [8,20,24,26,27]. According to *in vitro* experiments, the degradation of chitosan by lysozyme affords a certain yield of chitooligosaccharide [29]. These chitooligosaccharides play an important role in antimicrobial activity, tumor suppression, immunostimulation, wound healing, and tissue regeneration [14,30,31,32,33,34,35], consequently a chitosan with a lower DA and molecular weight might be better than one with a higher DA and molecular weight for the preparation of scaffolds for tissue engineering applications.

Chitosan quality depends on its biological source and the extraction procedure used [36]. Chitosans isolated from crustacean sources such as shrimp and crab shells and squid bone plates have a high molecular weight with low polydispersity, DA below 20% and a 1% solution viscosity of 500-1,700 cps, whereas fungal chitosan has a low molecular weight with high polydispersity, DA lower than 15% and a 1% solution viscosity of 10-15 cps [2,6,7]. However the molecular weight and DA of chitosans extracted from crustacean sources are variable, depending on the treatment conditions. Low molecular weight chitosans from crustacean sources have been produced from high molecular weight chitosan by physical treatments (like radiation and microwave irradiation), chemical treatments (including treatments with NaNO_2_, H_2_O_2,_ HNO_2_, phosphoric acid, HCl), and mechanical treatments (sonication, etc.) [32,37,38,39,40,41,42]. In the case of chemical treatment, the residual chemicals must be carefully removed from the low molecular weight chitosan for its safe use in medical applications [42]. Moreover, the residual protein in chitosan should be removed as completely as possible, as a small proportion of the human populations is sensitive to seafood proteins and will develop an allergic reaction in contact with those proteins. As a result, fungal chitosan, which has low molecular weight and DA, might be an attractive alternative for the preparation of tissue engineering scaffolds.

Chitosan is an important component of the cell wall of certain fungi; particularly the Zygomycetes class [3]. In the cell wall of fungi, chitosan consists of two forms: a free form and chitosan bonded to glucan [43,44]. The physico-chemical properties of chitosans extracted from different fungi have been characterized [4,5,43,44]. The quantity and quality of chitosan obtained from fungal cell walls depend highly on the fungal species involved, fermentation conditions and chitosan extraction procedures [2,4,5,43,44]. Among the various Zygomycetes fungi, *Gongronella butleri* gave the highest yield of chitosan [4,45]. Fungi can be grown by solid substrate and submerged fermentations, and the yield of chitosan obtained by solid substrate fermentation is higher than that obtained by submerged fermentation [2,5].

In this research chitosan was extracted from the mycelia of the fungus *Gongronella butleri* USDB 0201 grown in solid substrate fermentation and the extracted chitosan was used to prepare scaffolds for tissue regeneration templates. While a number of researchers have used chitosans obtained from shells of shrimps and crabs, and squid bone plates to prepare scaffolds for tissue engineering and studied the mechanical and biological properties of these scaffolds [20,22,25,46,47,48,49,50,51,52,53,54,55], up to now, no data on the application of fungal chitosan for the preparation of scaffolds for tissue engineering has been published.

Data on the effect of chitosan molecular weight on the characteristics of chitosan scaffolds has been published in a limited number of papers [24,27,28]. Most of the published research has used chitosan with molecular weight 10^5^-10^6^Da and DA 5-20% [20,22,25,46,47,48,49,50,51,52,53,54,55], so chitosans with molecular weight of 10^5^-10^6^Da and DA 10-20% obtained from shells of shrimps and crabs and squid bone plates were selected for the preparation of scaffolds and the mechanical and biological properties of those chitosan scaffolds were evaluated in parallel with the fungal chitosan scaffolds to observe the characteristics of scaffolds prepared using chitosans from different sources. Moreover, the biocompatibility of chitosan scaffolds such as cell morphology, attachment, proliferation and viability were also studied by cultivation of fibroblast NIH/3T3 cells on those scaffolds. Based on these observations, the advantages and disadvantages of fungal chitosan scaffolds for use as a tissue regeneration template are discussed in this report.

## 2. Results

### 2.1. Morphologies of chitosan scaffolds

**Figure 1:**
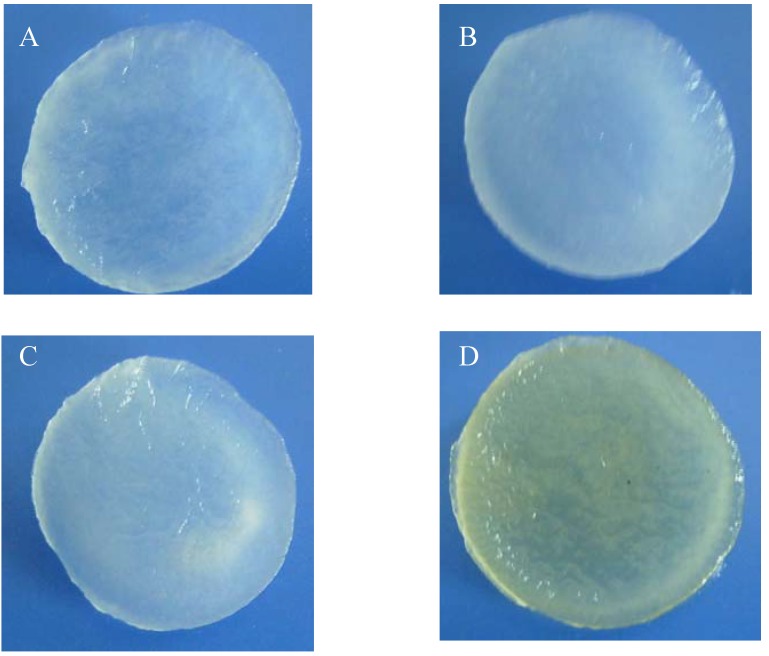
Photographs of SHCTS (A), CRCTS (B), SQCTS (C) and FCTS (D). All scaffolds were under wet conditions.

Chitosan scaffolds (CTS) were prepared using chitosans obtained from shells of shrimps (SHCTS) and crabs (CRCTS), squid bone plates (SQCTS) and fungal mycelia (FCTS) (Table 1). The neutralized SHCTS, CRCTS and SQCTS were colorless when wet and white in the dry form, while FCTS were pale yellow in both in the wet and dry form (Figure 1).

**Table 1:** Mechanical and biological properties of scaffolds prepared using chitosans obtained from shells of shrimps and crabs, squid bone plates and fungal mycelia.

Parameters			*Shrimp shells* *(SHCTS)*	*Crab shells* *(CRCTS)*	*Squid bone plates* *(SQCTS)*	*Fungal mycelia* *(FCTS)*
Size of scaff old (mm)	Before neutralization	Thickness	4.17 ± 0.16	4.84 ± 0.44	4.02 ± 0.38	4.56 ± 0.40
		Diameter	30.5 ± 0.4	30.3 ± 0.4	30.4 ± 0.4	30.9 ± 0.4
	After neutralization	Thickness	4.50 ± 0.40	3.25 ± 0.15	3.4 ± 0.70	4.70 ± 0.40
		Diameter	24.2 ± 1.0	25.9 ± 0.7	25.4 ± 0.9	28.1 ± 1.7
Pore size of chitosan scaffold (μm)			64 ± 20	77 ± 22	54 ± 17	84 ± 21
Amount of absorbed water in scaffold (g/g of scaffold)			36 ± 4	43 ± 3	46 ± 0	53 ± 2
Porosity of water absorbed scaffold (%)			88 ± 4	90 ± 3	96 ± 5	97 ± 1
Tensile strength of scaffold		Force (cN)	141 ± 37	88 ± 09	151 ± 31	209 ± 20
		Elongation (%)	15.1 ± 4.4	20.3 ± 4.2	5.4 ± 1.7	7.2 ± 1.5
Degradation of scaffold (%)			2 ± 0.8	5 ± 0.7	5 ± 0.5	11 ± 1.1

**Figure 2:**
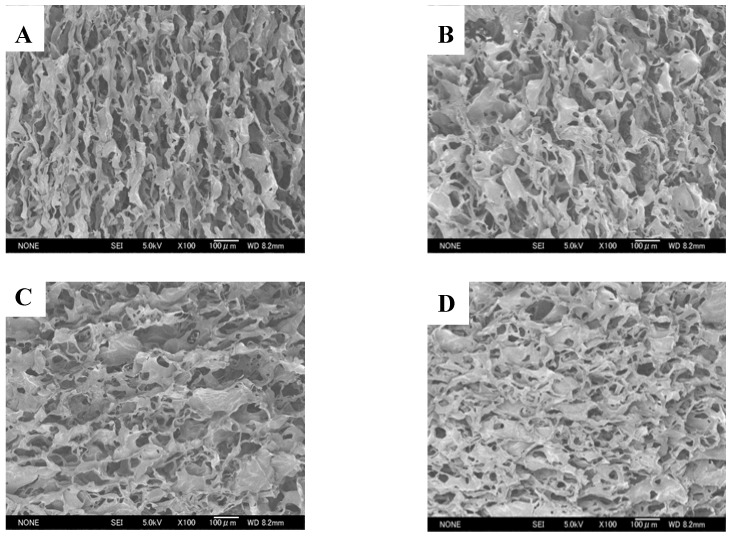
SEM micrographs of the cross-section of SHCTS (A), CRCTS (B), SQCTS (C) and FCTS (D).

All chitosan scaffolds were smooth, soft, sponge-like, flexible and strong enough to handle in wet and dry conditions without deformation. The diameter of all chitosan scaffolds after neutralization was about 16% smaller, when compared with the diameter of scaffolds before neutralization. The thickness of all scaffolds was similar except for the CRCTS (Table 1). Figure 2 shows Scanning Electron Microscopy (SEM) micrographs of cross-sections of the neutralized chitosan scaffolds. The SEM images show that 3D pore microstructures in all chitosan scaffolds were heterogeneous, with well-interconnected pores. Elongated pores were observed perpendicularly in the SHCTS, whose formation might be due to highly parallel ice crystal growth between the scaffold substrate layers, created by formation of hydrogen bonds between the long chain of polymers during the freeze-drying process. Polygonal pores, together with a small number of elongated pores, were observed randomly in SEM images of the CRCTS. Those different construction patterns of pores in the CRCTS might cause lower thickness in the scaffold during neutralization.

Polygonal pores were observed randomly in the SEM images of the SQCTS and FCTS. The scaffold substrate layers in all chitosan scaffolds were made up of sheet-like structures. A higher number of small sheet-like structures were observed in the scaffold substrate layer of the FCTS than that in the CRCTS and SQCTS ones. According to those observations, it can be assumed that FCTS had a higher level of connectivity of scaffold substrate layers than the others. The mean diameter of pores on all chitosan scaffolds was found in the range of 60-90 μm (Table 1, Figure 2).

### 2.2. Water absorption properties of chitosan scaffolds

The water absorption properties of chitosan scaffolds were studied by soaking them overnight in phosphate buffer (pH 7.4) at room temperature. The polygonal pores in the CRCTS, SQCTS and FCTS retained higher amounts of water than the elongated pores in the SHCTS (Table 1). Similar amounts of absorbed water could be observed in the CRCTS, with a small number of elongated pores, and in the SQCTS. The polygonal pores and a higher number of small sheet-like structures of scaffold substrate layers in the FCTS retained higher amounts of water, suggesting that not only polygonal pore morphology but also the small sheet-like structures in scaffold substrate layers supported the absorption of high amounts of water. The size and shape of water absorbed scaffolds were constant in neutral medium for more than three months. Consequently the porosity of scaffolds was examined after water absorption because the biological properties of scaffolds such as biodegradation and biocompatibility were studied on water absorbed scaffolds. The porosity of the FCTS was higher than that of the CRCTS and SQCTS (Table 1). Among them, the SHCTS has the lowest porosity (Table 1). This might be one of the reasons that the highest amount of water was absorbed by the FCTS than in others.

### 2.3. Mechanical properties of chitosan scaffolds

The mechanical properties of chitosan scaffolds were studied under dry conditions. The FCTS and SQCTS elongated only 5-7% when forces of 209 and 151 cN were applied, respectively (Table 1). On the other hand, the SHCTS and CRCTS, with elongated pores, stretched 15 and 20% under applied forces of 141 and 88 cN, respectively (Table 1). It was observed that although CRCTS and SQCTS have the same molecular weight and DA, the former was easier to deform. The FCTS was tougher and stronger than the others, which could be attributed to the small sheet-like structures of its scaffold substrate layers. 

### 2.4. In Vitro degradation of chitosan scaffolds

The degradation behavior of all scaffolds was studied *in vitro* by degradation with lysozyme. Table 1 compares the residual weight of chitosan scaffolds after degradation with lysozyme. All chitosan scaffolds retained a constant shape till the end of the degradation process. The SHCTS maintained 98% of its initial weight and the CRCTS and SQCTS retained 95% of their initial weight after 14 days of degradation. The FCTS retained only 89% of its initial weight, which indicated a much faster rate of weight decrease than that of the SHCTS, CRCTS and SQCTS. In the degradation process of chitosan scaffold by lysozyme, the enzyme must enter the scaffolds and react with the chitosan polymers. The SHCTS, CRCTS, SQCTS and FCTS all have pore sizes of about 60-90 μm, which provides a large enough area for lysozyme to enter, but the morphology of the scaffold substrate layers is different in each one. The FCTS, which has a higher number of small sheet-like structures in the scaffold substrate layers, thus has a larger surface area for lysozyme to access than other scaffolds, and consequently more chitosan molecules can be cleaved by lysozyme in the FCTS scaffold than in other scaffolds. In addition the pore morphology in the scaffolds affects their water absorption properties, which also facilitate the penetration of lysozyme into the scaffolds. The elongated pores in SHCTS absorbed the lowest amount of water among all scaffolds (Table 1), and as a consequence, the substrate layers of SHCTS will be less swollen than the others and will have less space for lysozyme to access the polymer chains.

### 2.5. Assessment of attachment, morphology and proliferation of Swiss mouse embryo fibroblast NIH/3T3 cells on chitosan scaffolds

**Figure 3:**
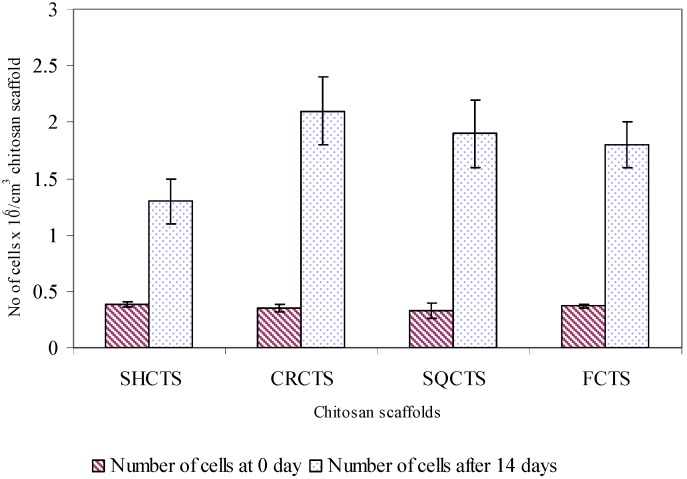
Attachment and proliferation of fibroblast NIH/3T3 cells on SHCTS, CRCTS, SQCTS and FCTS. Fibtoblast NIH/3T3 cells were cultured for 0-14 days on and in porous chitosan scaffolds at 37^0^C in a humidified 5% CO_2_ incubator.

Swiss mouse embryo fibroblast NIH/3T3 cells were cultured on SHCTS, CRCTS, SQCTS and FCTS and the cell attachment, morphology, viability and proliferation on these scaffolds was observed. The cell attachment and proliferation were evaluated using a direct cell count method. The percent of attached cells on all chitosan scaffolds was found to be greater than 90% (Figure 3) and the number of attached cells on all scaffolds was similar, which seemed to suggest that fibroblast cells could attach well on chitosan scaffolds with molecular weights of 10^4^-10^6^Da and 10-20% DA.

**Figure 4:**
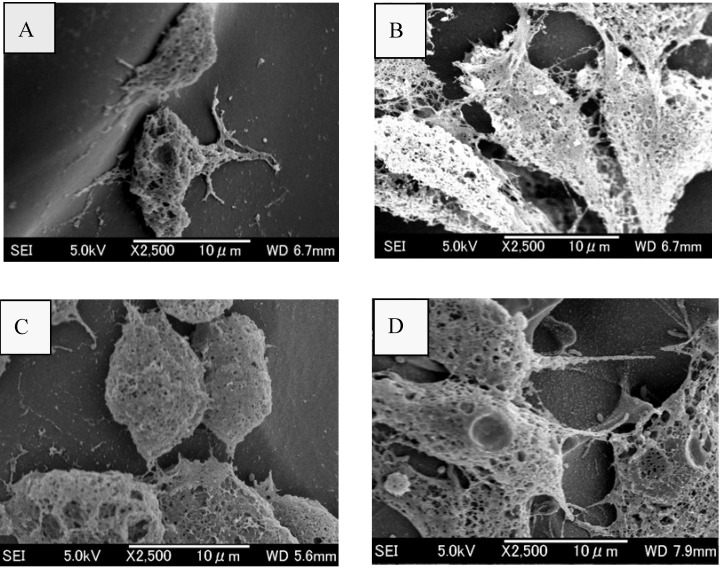
SEM micrographs of fibroblast NIH/3T3 cells grown on SHCTS (A), CRCTS (B), SQCTS (C) and FCTS (D) for 3 days.

**Figure 5:**
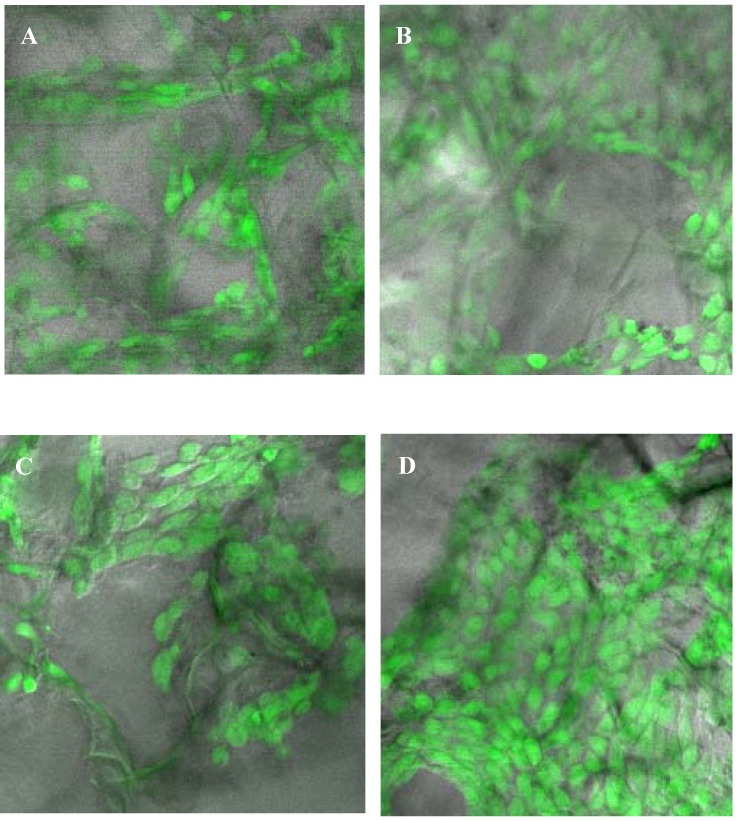
Confocal laser microscopic images of 14 days old fibroblast NIH/3T3 cells grown on SHCTS (A), CRCTS (B), SQCTS (C) and FCTS (D). Fibroblast cells were stained with fluorescein diacetate, FDA to stain viable cells green.

After cell attachment, the cell-seeded SHCTS, CRCTS, SQCTS and FCTS were incubated in DMEM medium at 37^o^C in a humidified 5% CO_2_ incubator for 3-14 days. Under the light microscope, the development of lamellipodia and filopodia of cells could be observed within one day of incubation (data not shown). The morphology of fibroblast cells on all scaffolds was studied by SEM after three days of cultivation (Figure 4). The cell viability on the scaffolds was observed by confocal laser scanning microscope (CLSM) after 14 days of cultivation (Figure 5), at the same time, proliferation of fibroblast cells on all scaffolds was also determined. Viable cells on all chitosan scaffolds displayed a normal polygonal morphology as well as a completely spread on all chitosan scaffolds (Figure 4 and Figure 5). The cells on CRCTS, SQCTS and FCTS were more flattened and more elongated than cells on SHCTS.

The randomly distributed fibroblast cells on all chitosan scaffolds were connected each other and formed a cell network across the surface of the scaffolds. The size of fibroblast cells is about 10-30 μm (Figure 4). In order to observe the viability and migration of fibroblast cells in the scaffolds, scaffolds were sliced into thin sheets (about 1 mm) with the help of surgical scissors and observed under a confocal laser scanning microscope. The polygonal morphology with live cells was observed on the top and bottom surfaces and on the middle surface of all scaffolds. Therefore it can be assumed that pore size, pore morphology and porosity of all scaffolds were sufficient for nutrients to enter for cell growth, to allow cells to migrate, to release out metabolic products produced from cells and to diffuse oxygen. Up to 14 days cultivation, no large differences in the morphology and viability of cells could be observed on all chitosan scaffolds.

**Figure 6:**
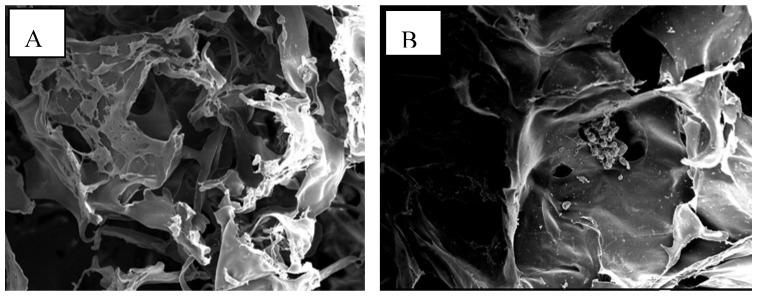
Fibroblast cells growth with polygonal morphology on the chitosan scaffold without air bubble trapped during neutralization (A) and cell with round morphology on the chitosan scaffold with air bubble trapped during neutralization (B).

On the other hand, it should be pointed out that scaffold preparation method is also a critical point to obtain a novel scaffold for growth of cells with the right morphology. Fibroblast cells on the chitosan scaffolds without air bubbles trapped during neutralization were found to have a normal polygonal morphology (Figure 6A) and spherical morphology with aggregated fibroblast cells was observed on the chitosan scaffold which had trapped air bubbles during the neutralization step (Figure 6B). Therefore, air bubbles must be removed completely from the scaffold during neutralization to obtain the complete neutralization on all the scaffold surface. The entrapped air bubbles in the scaffolds can be easily removed by neutralization of the scaffold immediately after freeze-drying, by loading NaOH to the scaffold in the horizontal direction or by using a vacuum pump while the scaffold is submerged in NaOH solution.

The proliferation of fibroblast cells on all chitosan scaffolds was studied after 14 days of cultivation. Figure 3 shows the number of fibroblast cells on all scaffolds for 0 and 14 days. It was found that about second to third generations of fibroblast cells were proliferated on all chitosan scaffolds. The number of proliferated cells on the CRCTS, SQCTS and FCTS was significantly higher than that on SHCTS. The latter, which has a high molecular weight, could retain a lower amount of water than the other scaffolds, therefore the CRCTS, SQCTS and FCTS absorbed more medium in the scaffolds than SHCTS. The cells on/in the scaffold will utilize the absorbed medium for their migration and as a nutrient for their growth. The cell migration is one of the important cellular activities for cell-cell and cell-extracellular matrix interactions, which plays an important role in the mechanism of cell proliferation on the matrix. This might be one of the reasons for the decrease in the number of proliferated fibroblast cells on the SHCTS.

## 3. Discussion

### 3.1. Mechanical and biological properties of chitosan scaffolds

In the process of chitosan production from chitin by concentrated NaOH, Itoh *et al*., observed that the aligned structure of chitosan molecules has no influence on removal of protein, calcium phosphate and amide groups from crab tendon chitin through a strong treatment in 50% NaOH at 100^o^C [51]. In the process of chitosan scaffold construction, firstly, chitosan powder was dissolved in acetic acid. In this process, the alignment of chitosan molecules will collapse and disappear. In the freezing process, rearrangement of polymer chains occurs during construction of interconnecting network pores and scaffold substrate layers by formation of hydrogen bonds between the polymer chains, water molecules and polymer chains and water molecules. Therefore, the pattern of rearrangement of polymer chains in the scaffold substrate layers might depend on the degree of acetylation, molecular weight and charge distribution in the polymer chains and original crystalline forms of each chitosan. Different patterns of pores and scaffold substrate layers could be observed in the SEM images of SHCTS, CRCTS, SQCTS and FCTS (Figure 2). Elongated pores were observed in the SHCTS and CRCTS and polygonal pores were observed in the CRCTS, SQCTS and FCTS. Here, the DA of SHCTS, CRCTS, SQCTS and FCTS was nearly the same, but the molecular weight and charge distribution in the polymer chains were different in each scaffold. Moreover, the original crystalline form of SHCTS and CRCTS is the α-form and for SQCTS it is the β-form [56], whereas FCTS lacks any alignment structure and the polymer chains are synthesized directly in the hyphae of fungus *Gongronella butleri* in a random manner and linked to glucan polymer. It can be concluded that chitosan with high molecular weight formed elongated pores and the medium to low molecular weight chitosan polymers formed polygonal pores. In 2006, Amaral *et al*. observed a more homogeneous pore distribution with the absence of fiber-like structure in squid chitosan scaffolds with molecular weight 10^5^Da and 4-30%DA and a more heterogeneous pore distribution with the presence of fiber-like structures in squid chitosan scaffolds with molecular weight 10^5^Da and 49%DA [25]. Thein-Han and Kitiyanant reported that the pores of shrimp chitosan scaffold with 5-12%DA were smaller than the pores of 30% DA scaffolds [20].

The pore size, porosity and distribution pattern of pores in the scaffold affect the water absorption and water vapor permeability of the scaffold [20,23,54,58,59,60,61,62,63,64]. In addition, the neutralization medium also should be considered to obtain optimal water absorption of chitosan scaffolds because about 1,500-2,000% water was absorbed by shrimp chitosan scaffolds (molecular weight 8.1 x 10^5^ Da and 5-12% DA) neutralized with ethanol [20], whereas SHCTS, CRCTS, SQCTS and FCTS (molecular weight 10^4^-10^6^ Da and 10-20% DA) neutralized with 1 M NaOH absorbed more than 3,500% water (Table 1).

Moreover pore size, porosity and distribution pattern of pores in the scaffold highly influence on the mechanical properties of scaffolds [20,57,65,66]. Hsu *et al*. reported that chitosan membranes with high molecular weights led to higher mechanical strength than membranes with low molecular weights [28]. They used chitosans with similar DAs. According to our results, the mechanical properties of chitosan scaffold with lower molecular weight were higher than that of chitosan scaffold with higher molecular weight. The reason might be the difference in the construction of chitosan membrane and scaffold. In the construction of chitosan membrane, chitosan polymer chains did not rearrange again during the evaporation of solvent. For the construction of scaffolds, chitosan polymer chains rearranged again during the freezing process and formed the pore and scaffold substrate layers by removing the ice crystal during the freeze-drying process.

Another important property of chitosan scaffolds for use as a tissue regeneration template is the gradual degradation of the scaffold within the body. Degradation of chitosan matrixes has been studied *in vitro* and *in vivo* models by many researchers [8,18,20,24,26,29,37,51,55,67,68,69,70,71,72,73,74,75]. According to the results obtained from *in vitro* experiments reported by different authors, chitin/chitosan polymers could be degraded with the action of lysozyme, which is present in the body fluid and chicken egg white [20,29,76,77,66,75]. Lysozyme recognizes N-acetyl glucosamine sequences in the chitin/chitosan molecules, thus lysozyme digestibility increases with increasing degree of N-acetylation in the polymer chain [29]. The chitosan matrix with high DA broke to pieces after a few days of lysozyme treatment and the matrix with low DA remained relatively constant for a long time [20,28,69]. In comparison, Thein-Han and Kitiyanant [20] reported that the remaining weight of shrimp chitosan scaffolds, which have molecular weight 8 x 10^5^Da and DA 12%, was about 90-80%, which was much faster than our experiment results. They used 500 μg/mL of lysozyme and we used only 10 μg/mL of lysozyme solution in our experiments and the rate of degradation of chitosan scaffolds by lysozyme is faster in the presence of higher amounts of lysozyme in the degradation medium. Moreover, the pore parameters of chitosan scaffolds could significantly influence their degradation behaviors [55]. According to our results, the FCTS scaffold showed higher lysozyme degradation rate than other chitosan scaffolds. It can be assumed that chitosan with lower DA and lower molecular weight was better than chitosan with higher DA and higher molecular weight for the preparation of a scaffold to use as a tissue regeneration template, for which the degradation period of the implanted chitosan scaffold should not be too long, i.e., the degradation rate of a scaffold must be optimal to maintain a stable structure long enough to obtain cell layers on the scaffold. The degradation of chitosan scaffolds by lysozyme gave monomers and oligomers of chitosan (data not shown). A similar observation had been reported by many researchers [29,66,78]. Many researchers have studied the physiological effects of glucosamine and N-acetylglucosamine on hard tissue forming cells and skin cells respectively [70,71,72,73,74], but little is known about glucosamine metabolism.

### 3.2. Biocompatibility of chitosan scaffolds

Proliferation of mammalian cells on a particular matrix takes place in three stages: first the cells attach on the matrix, then they spread and finally they divide in the presence of nutrients [22]. There was no significant difference in the number of fibroblast cells attached on SHCTS, CRCTS, SQCTS and FCTS. A similar observation has been reported by Tangsadthakun *et al.* [27]. Therefore cell attachment on the chitosan scaffolds has no correlation with the molecular weight or source of the chitosan and pore parameters. It should be noted that all chitosans used in this research had low DA, about 10-20%. Considering that cell attachment on the surface of chitosan scaffolds involves the binding of negative groups on the cell surface to the positive charges on the chitosan scaffold surfaces [22,52], the number of attached cells on a chitosan matrix is highly dependent on the DA of the chitosan and the type of cell line [22,27,52,79].

According to the results obtained from the studies on the attachment of different cell lines on chitosan matrix by Hsu *et al*. [28] and Chatelet *et al*. [80] the number of attached cells on a chitosan matrix increased in the order of chondrocyte > fibroblast > keratinocytes. They assumed the negative charge density on the surface of chondrocyte > fibroblast > keratinocytes, because a negative surface charge on the cells preferred to attach on the positive charge surface on the chitosan matrix. In the same way, a chitosan with a low DA provides more cationic sites, which helps give a stronger electrostatic interaction with the negative charges on the cell surface [22,28,80]. Thus, the number of attached cells was similar on the chitosan matrixes with lower range of DAs, which showed higher number of attached cells than on chitosan matrixes with higher range of DAs [20,22,25,28,80]. Based on the published data, together with our results, it can be pointed out that not only the DA of chitosan in a lower range, molecular weight of chitosan, and source of chitosan, but also the characteristics of chitosan scaffold such as pore morphology and orientation and water absorption properties do not influence the attachment of cells on chitosan scaffolds.

Amaral *et al*. reported that the DAs of chitosan play an important role on the morphology of attached cells [25]. They observed that MG-63 osteoblast-like cells from human osteosarcoma attached and spread with long filopodia and numerous cell-to-cell contacts on chitosan scaffolds with lower DA (13%), but cells tended to remain spherical and to grow into spheroid-like cellular aggregates on chitosan scaffolds with higher DA. Similarly, endothelial cells [18], smooth muscle cells [18], buffalo embryonic stem-like cells [20], and Neuro-2a cells [54] attached and proliferated with normal morphology on chitosan scaffolds with low DA (10-15%). Although low DA chitosan scaffolds were used to culture mesenchymal stem cells [16], fibroblast cells [17] or human umbilical vein endothelial cells [19], the cells were found to have a round morphology. According to the report of Ma *et al*. [52] human neofetal dermal fibroblasts could grow and proliferate with an almost extended shape, a little like in normal monolayer tissue culture on the flat bottom of some of the large pores of a bilayer scaffold with DA 26.5%, but the morphology of fibroblast cells remained spherical on a rough surface such as on the pore wall, at the edges of small pores and shallow pores. The cell spreading can be considered as a sign of healthy attachment on the supporting scaffold and round morphology can have many interpretations. It could be that the cells tend to be round by themselves or that they do not spread due to unfavorable metabolic conditions or an incompatible scaffold surface. Cell adhesion molecules are found on the surface of all cells and play a role in cell–cell and cell–extracellular matrix interactions [81]. Therefore, one should consider that not only the overall charge density of the chitosan (DA) is important, but also the distribution of the charge along the molecule because each cell line expresses a different type and level of cell adhesion molecules in specific environments [82]. Therefore DA of chitosan and charge distribution on the chitosan polymers should be considered to grow cells with the right morphology on a chitosan scaffold. Rounded cell morphology is considered as a sign of poor attachment to the substrate, forming less focal adhesions and consequently would correspond to a lower degree of cell proliferation and differentiation [76,83].

According to the results obtained from our research and Tangsadthakun *et al*. [27], the proliferation of fibroblast cells on chitosan scaffold with low molecular weight was higher than on chitosan scaffold with high molecular weight. On the other hand, it should be pointed out that chitosan with lower DA stimulated higher cell proliferation than chitosan with higher DA [20,25,28,77,80]. Hsu *et al*. [28] assumed that the steric barrier due to the longer polymer chain inhibited the charge effects, as a consequence a decrease in proliferation of cells was observed on chitosan matrices with high molecular weight and lower DA. Therefore chitosan with lower molecular weight and lower DA is better than chitosan with higher molecular weight and lower DA for the construction of scaffold for tissue engineering since fibroblast NIH/3T3 cells and L929 mouse fibroblast cells [27] could attach and proliferate faster on chitosan scaffolds with low molecular weight and DA.

Hsu *et al*. [28] and Chatelet *et al*. [80] observed that chitosan films could support the proliferation of chondrocyte and keratinocytes, but fibroblast cells did not proliferate on these films. In contrast, Ma *et al*. [52] observed the proliferation of fibroblast cells on the bilayer structure of chitosan film and sponge. According to our results and data in the reports of Tangsadthakun *et al*. [27] and Ma *et al*. [52], it can be concluded that fibroblast cells could attach and proliferate well on chitosan scaffolds.

Nevertheless the growth rate of fibroblast cells on the CRCTS, SQCTS and FCTS (only 5-6 fold after 14 days of cultivation) was slower than the growth rate of buffalo embryo stem-like cells on chitosan scaffold (about 10-fold within 14 days) [20]. There are a lot of reasons on the decrease in growth rate of cells on chitosan scaffold:

(1) Thickness of scaffold, i.e., the cell growth rate decreased when the thickness of scaffold increased [46],
(2) Longer the lag phase during growth of cells on chitosan scaffold [22],
(3) High or low number of cells inoculated to the scaffold [22], i.e., low inoculum concentration resulted in cellular stress during growth of the cell on the chitosan scaffold and high initial cell concentration resulted in slower growth rate, and
(4) Interaction between the cell and scaffold surface [28].

In conclusion, attachment, morphology and proliferation of cells on chitosan scaffold highly depend on type of cell lines, source and characteristics of chitosans, methods of chitosan scaffold preparation and characteristics of the chitosan scaffolds. In addition, chitosan with low molecular weight and low DA has excellent potential as a scaffolding material for a variety of tissue regeneration systems.

## 4. Experimental Section

Shrimp chitosan (MW 2 x 10^6^ Da, DA 12%) was obtained from Bioprocess Technology, Asian Institute of Technology, Bangkok, Thailand. Crab chitosan (MW 1 x 10^5^Da, DA 20%) was obtained from Koyo Chemical Co., Japan. Squid chitosan (MW 2 x 10^5^Da, DA 15%) was obtained from squid bone plates obtained in Thailand. Swiss mouse embryo fibroblast NIH/3T3 cell line was purchased from Invitrogen, Japan. The culture medium for fibroblast cells was Dulbecco’s Modified Eagle Medium (DMEM, Gibco, Rockville, MD, USA) supplemented with 10% (v/v) fetal bovine serum (Gibco), 0.45% (w/v) glucose, 0.22% (w/v) NaHCO_3_ and 1% (v/v) penicillin/streptomycin solution (Gibco). Trypsin–EDTA solution (0.5% (w/v) trypsin with EDTA-4Na) was also purchased from Gibco. The water used was double distilled water obtained from the RFD 240NA water distillation apparatus (Advantec, Model RF 100170, Toyo Seisakusho Kaisha, Ltd., Japan). All other chemicals were an analytical grade purchased from Wako Chemical Co. (Japan) and were used without further purification.

### 4.1. Extraction of chitosan from cell wall of fungus Gongronella butleri USDB 0201 grown in solid substrate fermentation

*G. butleri* USDB 0201 (class Zygomycetes) was obtained from the Department of Biological Sciences, National University of Singapore. To obtain a high yield chitosan, the fungus was grown on peeled sweet potato pieces supplied with mineral solution and urea in a tray type solid substrate fermenter under optimal conditions [84,85]. The design of the fermenter is easy to operate and is suitable for scaling up with minor investment. After 7 days, the myceliar mass was harvested, washed with water and dried at 45 ^o^C. In order to obtain high yield chitosan from fungal mycelia, the conditions for the extraction of chitosan from fungal mycelia have been optimized and the extracted chitosan has been characterized by elementary analysis, IR and ^13^C-NMR spectroscopy [43,44,45,86]. Briefly, dried mycelia (1 g) were treated with 11 M NaOH (10 mL per g mycelia) at 45 ^o^C for 13 h. The alkali insoluble material (AIM) was collected and washed with distilled water to neutral pH and then dried. The dried AIM was suspended in 0.35 M acetic acid at 95 ^o^C for 5 h and treated with Termamyl, Type LS (Novo Nordisk, density 1.2 g/mL, activity 120 Kilo Novo alpha-amylase Units per gram of enzyme) in a shaking water-bath at optimal conditions: 4% (v/v) enzyme, 65 ◦C, 200 rpm for 3 h. The enzyme treated suspension was centrifuged at 1,600×*g* for 15 min and collected the chitosan solution and then adjusted the solution pH to 8–9 with 1M NaOH to precipitate the chitosan. The precipitate was collected by centrifugation at 1,600×*g* for 15 min and treated again with 1M NaOH to remove the enzyme from the chitosan. After that, chitosan precipitate was washed with distilled water to neutral pH and freeze-dried. The yield of chitosan that could be extracted from the fungal mycelia was 9-10 g/100 g mycelia. Fungal chitosan has molecular weight 5 x 10^4^Da and DA 13%.

### 4.2. Preparation of porous chitosan scaffolds

According to the published reports, larger pore size chitosan scaffolds could be constructed by freezing the chitosan solution at a slow rate, but at a higher freezing temperature, –20^o^ C [13,53]. Therefore in this research, chitosan powder (1g) was dissolved in 0.35 M acetic acid (100 mL), which is a common solvent to dissolve all chitosans, at room temperature for 1-2 days until the solutions became viscous, transparent and homogeneous. Each chitosan solution (8 mL) was transferred to a cultivation dish (diameter 35 mm) and frozen at –20 ^o^C and then freeze-dried.

After freeze-drying, the acetate molecules are in solid form in the scaffold cavities, as ions bound to the cationic amine groups in the chitosan. When the acetate is not removed or neutralized, the scaffold will swell rapidly and ultimately dissolve upon rehydration in a neutral aqueous medium [13]. In order to prevent the chitosan scaffold dissolution, the chitosan scaffolds can be rehydrated in ethanol-water mixtures using a solvent series with increasing water content or in NaOH solution [8,13,16,18,19,20,25,52,79,87,88]. Here, the physico-chemical properties of chitosan such as the degree of acetylation affect the shape of scaffold during cultivation when ethanol was used to wash out acetate [8,13,18,20,87]. In case of NaOH neutralization, the right NaOH concentration must be applied to maintain the shape and volume of chitosan scaffold during the cell culture period [13,52,79,88].

**Figure 7:**
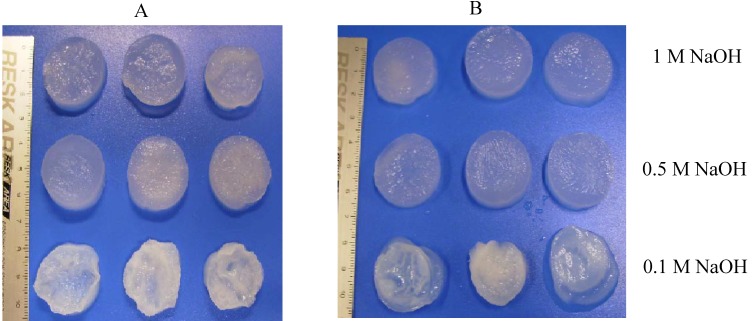
Morphology of crab chitosan scaffolds after neutralization with 1, 0.5 and 0.1 M NaOH in distilled water (A) and in 70% ethanol (B).

Crab chitosan scaffolds were neutralized in 10 mL of 0.01, 0.1, 0.5 and 1 M NaOH dissolved in distilled water or 70% ethanol (Figure 7) to determine the right NaOH concentration for the neutralization of chitosan scaffolds. It was observed that scaffolds completely dissolved in 0.01 M NaOH in distilled water or in 70% ethanol solution during 5 h. The aggregation of scaffold cavities could be observed in scaffold neutralized in 0.1 M NaOH in distilled water or in 70% ethanol solution. Porous chitosan scaffolds could be obtained using 0.5 and 1 M NaOH in distilled water or in 70% ethanol solution as neutralized media. The best neutralization medium is 1 M NaOH dissolved in distilled water or 70% ethanol.

Here, air bubbles trapped inside the scaffolds was observed in the freeze dried scaffolds when the scaffolds were stored for 1 to 2 weeks and then neutralized with 1 M NaOH. The reason might be the moisture in the air will bind to acetate ions and amino groups on the surface of the scaffold. As a consequence, the surface tension of the scaffold will decrease and trapped air remains trapped for a long time during neutralization. Therefore immediately, freeze-dried chitosan scaffolds were neutralized with 10 mL of 1 M NaOH solution in order to prevent air trapping inside the scaffold during neutralization. After neutralization, scaffolds were thoroughly washed with double distilled water up to neutral pH. Before and after neutralization, the diameter and thickness of the dried scaffolds were measured with the help of a vernier.

### 4.3. Observation the morphologies of chitosan scaffolds

Wet neutralized chitosan scaffolds were sliced into rectangular shapes and then freeze-dried after soaking in water. The freeze-dried samples were mounted on sample stubs and coated with an ultra thin layer of palladium in a coating apparatus and then the pore morphology of the scaffolds was observed under a scanning electron microscope (JEOL- JSM 6700, Japan) at an accelerating voltage of (5 kV) and current of 10 μA. At the same time, the diameter of pores in each sample was measured using the JEOL- Image software. The total number of pores analyzed for each sample was 12. The values were expressed as the mean value of twelve independent replicates.

### 4.4. Study the water absorption property of chitosan scaffolds

The water absorption properties of neutralized chitosan scaffolds were determined according to the method described by Mao *et al*. [46] with minor modifications. Dried scaffold with a known weight (W_i_) was soaked overnight in phosphate buffered saline (PBS buffer, 8 g NaCl, 0.2 g KCl, 1.44 g Na_2_HPO_4_ and 0.24 g KH_2_PO_4_ per liter, pH 7.4, 7 mL ) at room temperature. After that sample was removed from buffer solution, the wet weight of the scaffold (W_w_) was measured. The weight of water absorbed in scaffold (W_a_) was calculated as a fraction of dry weight of the scaffold as shown in equation 1. The values were expressed as the means value of three independent replicates.

W_a_ (g water/g of scaffold) = (W_w_ – W_i_)/W_i_ (1)

### 4.5. Determination of the porosity of water absorbed chitosan scaffolds

Dried chitosan scaffold was soaked overnight in water. Three pieces of tissue paper were dried overnight in an oven at 55^0^C and then the weight of tissue papers (W1) was measured. The water absorbed scaffold was taken from the water medium and the diameter and thickness of the scaffold were measured and the volume of the water absorbed scaffold, V1 = (∏r^2^h) then calculated. The water absorbed scaffold was placed on the top of the tissue papers in a centrifuge tube and then centrifuged at 4,500 rpm for 5 min. After that scaffold was removed from the tissue papers and the weight of wet tissue papers (W2) was measured and then the weight of water in the void space of scaffold, W3 = (W2 –W1) was calculated. The volume of water in the void space, V2 was determined by dividing the weight of water in the void space of scaffold, W3 by the density of water (1.0) (Equation 2)

Porosity of water absorbed scaffold (%) = (V2/V1) x 100
(2)

### 4.6. Examination the mechanical properties of chitosan scaffolds

Neutralized chitosan scaffolds were cut with scissors into strips (dimensions = 5 x 5 x 10 mm^3^) in wet condition to prevent deformation of the samples and then the scaffold was freeze-dried after by soaking in water. The dried sample was sandwiched between two pieces of sandpaper on either side of scaffold with the help of Scotch permanent adhesive glue. About 2.5 mm of the scaffold strip was glued on either side to the end of the paper pieces. The tensile properties of scaffolds were examined with an Orientec StA-1150 (Japan) at room temperature. The guage length between the two grips was set at 10 mm and the speed of testing was set at 10 mm/min. Sand papers were attached to both sides of the grips. The end of each grip was the beginning of the attached sample in order to prevent the deformation of samples during mounting. The applied force and elongation at break were determined. The values are expressed as the mean of five independent replicates.

### 4.7. In vitro degradation of chitosan scaffolds

The biodegradation rate of the neutralized chitosan scaffolds was determined *in vitro* by measuring the change in sample weight over time under treatment with lysozyme under specific conditions. The initial dry weight of scaffolds was determined and recorded as W1. The size of the scaffolds was the same as the size of the scaffold after neutralization (Table 1). The scaffolds were sterilized in PBS buffer at 121°C for 15 min. After cooling, free solution was removed from the sterilized scaffolds and incubated in 7 mL of sterilized PBS buffer containing 10 μg/mL hen egg white (HEW) lysozyme. All incubations were done in six well plates at 37°C in a humidified 5% CO_2_ environmental incubator. Media were replaced weekly with freshly prepared lysozyme solution. After 14 days of incubation, the samples were removed from the degradation media, washed with distilled water and freeze-dried. The weight of the freeze-dried scaffolds was recorded as W2. The percentage degradation weight of the scaffolds was calculated using Equation 3. The values were expressed as the mean value of three independent replicates.

Degradation weight (%) = (W1 –W2)/W1 x 100
(3)

### 4.8. Attachment and proliferation of fibroblast NIH/3T3 cells on chitosan scaffolds

Neutralized chitosan scaffolds were cut into rectangular test samples (5 x 5 x 5 mm^3^) and placed in test vials containing distilled water (2 mL) and then sterilized in an autoclave at 121°C for 15 min. After cooling, free water was removed from the sterilized scaffold and scaffold was seeded with 150 μL of cell suspension (3.29 x 10^5^cells/mL) on and in the scaffold with the help of micropipette. Cells were allowed to attach on and in the chitosan scaffolds at 37 ^o^C in a humidified 5% CO_2_ incubator for 4 h. After 4 h incubation, the cell-seeded scaffold was washed with DMEM medium (1 mL) three times and the washing medium collected and then centrifuged to precipitate the unattached cells. The precipitated cells were collected by decanting the washing medium and the cell pellets were re-suspended in 100-150 μL of DMEM medium. After that the number of unattached cells in the suspension was counted with a hemocytometer and the volume of the cell suspensions measured with the help of micropipettes. The number of attached cells on the chitosan scaffolds was estimated by subtraction the number of unattached cells from initial number of cells in the inoculum.

The cell-seeded scaffolds were incubated in DMEM medium (5 mL) at 37 ^o^C in a humidified 5% CO_2_ incubator. Fresh medium was provided every 1-3 days depending on the incubation period. The number of proliferated cells was counted after 14 days of incubation. Prior to cell counting, scaffolds were washed three times with PBS buffer to remove unattached cells and FBS from the medium. The scaffolds were scratched into small pieces with the help of a surgical scissor and immersed in 2 mL of 1 x trypsin-EDTA aqueous solution and then incubated at 37^o^C for 10 min to detach the cells from the pieces of scaffolds. The suspension was then sheared mildly with a micropipette to detach the cells from the scaffold material and counted the number of proliferated cells using a hemacytometer.

### 4.9. Observation the morphology and viability of fibroblast NIH/3T3 cells on chitosan scaffolds

For cell morphology, 3 day-old fibroblast cells on chitosan scaffolds were washed three times with PBS buffer and once with distilled water. After that, cells on scaffolds were dehydrated in a graded ethanol solutions (15%, 25%, 35%, 45%, 70% and 95% ethanol) each for 10-20 min and then freeze-dried. Samples were attached to sample stubs and coated with ultra thin layer of palladium in a coating apparatus and then observed morphology of cells by scanning electron microscopy (JEOL- JSM 6700, Kyoto, Japan). To assess cell viability, 14 day-old cell cultured chitosan scaffolds were washed three times with PBS buffer and incubated in 1 mL of 2 μg fluorescein diacetate/mL solution (FDA, Wako Pure Chemicals, Japan) dissolved in PBS buffer at 37^o^C for 10 min to stain viable cells green. After 10 min, the samples were viewed under a confocal laser scanning microscope (Carl Zeiss Laser Scanning Microscopy, Axiovert 200 M, LSM5PASCAL, Germany).

## 5. Conclusions

The application of chitosan as a scaffolding material in tissue engineering has a lot of advantages over other biopolymers; (1) morphology of pores and mechanical properties of scaffold can be easily controlled using specific chitosans, (2) scaffolds are stable in size and shape during cell culture period, (3) scaffolds can maintain enough medium to grow cells with right cellular activities, finally, (4) scaffolds can be degraded by lysozyme. However, chitosan is not a unique polymer. The physico-chemical properties of chitosans depend on their biological sources and the extraction procedures used. These properties inherently affect the size and morphology of pores in scaffolds, which consequently affect on the water absorption, mechanical properties, biodegradation properties and cellular activities of the scaffolds. Among the tested chitosans in here, fungal chitosan is an excellence chitosan for the preparation of scaffold for tissue engineering since it showed better water absorption, lysozyme degradation, mechanical properties and cellular activities than shrimp, crab and squid chitosan scaffolds. Moreover, the application of fungal chitosan in medical sector has a lot of advantages when compared with crustacean chitosans such as the degree of acetylation and molecular weight of fungal chitosan are more stable than crustacean chitosans, fungal chitosan is free of shrimp proteins and thereby avoids the shrimp allergy problems. In addition, fungal mycelia production and enzymatic chitosan extraction method also have been developed to obtain high yield fungal chitosan in very easy way and can be developed for the large-scale production of fungal chitosan. Therefore fungal chitosan is proposed as a most suitable chitosan for the preparation of scaffolds for tissue engineering.
